# The role of subsurface geomechanics in the green energy transition

**DOI:** 10.1098/rsos.241516

**Published:** 2025-05-07

**Authors:** Adriana Paluszny, Robert W. Zimmerman

**Affiliations:** ^1^ Earth Science and Engineering, Imperial College London, London, UK

**Keywords:** subsurface, geomechanics, energy, green transition, induced seismicity, renewable energy

## Abstract

The global energy landscape is currently experiencing a significant shift towards non-hydrocarbon, sustainable energy sources, often referred to as ‘green energy’. This transition is being driven by the urgent need to address the problem of global warming caused by greenhouse gases, most of which are generated by the burning of fossil fuels. This article provides an overview of the role that subsurface geomechanics will play in this transition, focusing on green energy technologies such as carbon sequestration, geothermal energy production, hydrogen storage and nuclear waste disposal. The article starts with a review of geomechanical properties and structures that will be relevant to the green energy transition, such as *in situ* stresses, elastic moduli, strength properties, permeability, faults and fractures. This is followed by introductions to the four green energy technologies mentioned above. The next section focuses on the specific geomechanical challenges associated with each of these technologies, such as surface subsidence, induced seismicity and fluid and contaminant leakage. Gaps in existing knowledge, and potential pitfalls to be avoided, are highlighted. The article concludes with a brief discussion of public perception of environmental risks associated with subsurface energy technologies. It is concluded that geomechanics will play a key role in each of these emerging subsurface energy technologies, and the knowledge and tools that have mainly been developed in the context of fossil fuel exploitation will be key to these developments.

## 1. Introduction

The global energy landscape is currently experiencing a significant shift, driven by the urgent need to address the problem of global warming caused by greenhouse gases, most of which have been generated by the burning of fossil fuels [1]. In the following discussion, the term ‘green energy’ will refer to energy systems that seek to mitigate climate change by reducing carbon emissions, while supporting sustainable long-term, large-scale renewable energy. This transition to green energy is widely acknowledged by policymakers, industry stakeholders and the public, emphasizing the importance of moving towards sustainable and renewable energy alternatives. This shift presents challenges such as energy security, short- and long-term energy storage, decarbonization of hard-to-abate industries and the low-carbon delivery of minerals and metals essential to delivering the infrastructure required for the set-up and deployment of low-carbon and renewable energy solutions, seeking to avoid climatic tipping points [2]. This transition period notably includes the electrification of chemical industrial processes [3], as well as the decarbonization of the cement, steel and aluminium industries, blue hydrogen production and waste management [4], through multiple strategies including subsurface carbon sequestration at the gigatonne scale [1]. This will require the rapid acceleration of our capacity to store CO_2_, currently at the megatonne scale, requiring a significant and sustained growth of the industry within a relatively short period, which will involve the injection of CO_2_ at a scale similar to that which the hydrocarbon industry currently undertakes [5]. The transition will also require the use of massive battery electricity storage [6] and the use of alternative fluids as energy carriers, such as hydrogen [7], ammonia [8], methanol, dimethylether and methylcyclohexane [9]. These alternative fluids are likely to be stored in porous media at the equivalent terawatt-scale, but still face uncertainties on the impact of cycling of caprock integrity and leakage control, flow properties and management of microbial degradation of the fluids [10].

These factors, compounded with an uncertain geopolitical future, spatio-temporal challenges posed by climate change, and fluctuations in economic stability and regional market volatility, play important roles in shaping the development of sustainable and long-term low-carbon solutions that will become the new backbone of the energy future. To address these challenges, solutions need to be effective on a large scale, economically viable and environmentally sustainable for a variety of geographical locations. The subsurface plays an important role in delivering a sustainable pathway for these solutions, and our ability to engineer sustainable solutions that responsibly utilize the subsurface to these means will be a significant factor in realizing this future.

This review article aims to provide a comprehensive overview of the role of subsurface geomechanics in supporting the green energy transition. It highlights the most up-to-date science in this field, and the advances that have been made to understand how the subsurface can be utilized, but also modified, to achieve these goals. The article starts by describing the key concepts that relate to geomechanical properties and processes of the subsurface, highlighting why these are relevant to the green energy transition. This includes describing key mechanical and transport properties of rocks, fractures, subsurface structures such as faults and fractures, and processes such as subsidence and induced seismicity, while also discussing the heterogeneities and uncertainties inherent in subsurface systems. The article subsequently examines a range of green energy technologies, including carbon sequestration, geothermal energy, hydrogen storage and nuclear waste disposal, and explores how they are linked through the relevance of subsurface geomechanics to their implementation. The article also identifies common, persistent geomechanical challenges faced in the deployment of green energy projects and emphasizes the importance of comprehensive risk assessment and regulatory oversight. Many of these challenges are common to each of the aforementioned technologies and are linked to our ability to control the subsurface and our ability to modify its properties to aid the objectives at hand. Finally, the article explores the environmental impacts and societal perceptions surrounding green energy technologies, considering factors such as public acceptance and regulatory frameworks around common phenomena linked to these technologies.

## 2. Elements of subsurface geomechanics

### 2.1. Introduction

The role of subsurface rocks in the green transition is manifold. Rocks serve as a secure, remote repository for fluids and solids, with a vast capacity for storing huge volumes deep underground. Over the coming decades, the subsurface will acquire a series of new roles: sequestering hard-to-abate CO_2_ emissions and nuclear waste, storing energy in the form of compressed air, hydrogen and thermal fluids and providing mineable heat for sustainable regional heating and electricity production. In planning these operations, material properties and *in situ* conditions become key components of system understanding, used primarily in mathematical and numerical models to make predictions of the deformation and flow of rocks and fluids over long periods of time [11,12]. This section describes some of the newest findings in the description of rock transport properties, mechanical deformation and strength properties, and important discontinuities such as fractures and faults, and also discusses new developments in the understanding of their heterogeneity, and how these properties are measured in the field. Also discussed will be monitoring strategies and the variation of properties across spatio-temporal scales. *In situ* stresses and mechanical rock properties of subsurface rocks influence the deformation of the subsurface, and their measurement and understanding are instrumental for the prediction of subsurface behaviour during injection and withdrawal of fluids [13], as well as for the safe and sustainable construction and operation of wells [14,15], tunnels and shafts [16], which are all relevant in the context of large-scale subsurface operations. The properties described in this section are also of importance in the context of fracture growth for hydraulic stimulation [17,18], which is relevant in the context of *in situ* enhancement of permeability relevant to deep geothermal energy (DGE) systems [19], and fluid injectivity in the context of subsurface storage [20]. In particular, within the context of green energy development, economies of scale and societal acceptability become of increasing importance, with geomechanical events such as subsidence, leakage of fluids and induced seismicity playing an important role in facilitating the development of these technologies on a large scale. Economies of scale can facilitate lower costs and trigger tipping points that may substantially contribute to the acceleration of widespread adoption of clean energy [21].

### 2.2. Subsurface stresses

Subsurface rocks are always subject to stresses, caused by factors such as the weight of the overlying rock strata and lateral tectonic forces. Stresses are forces per unit area and consequently have units of N m^−2^ or Pascals. In general, the stress is represented by a 3 × 3 matrix (second-order tensor), the diagonal components of which represent *normal stresses* that act perpendicular to the surface, and the off-diagonal components of which represent *shear stresses* that act tangential to the surface. Typical notations for the stress components are *σ* and *τ*. A fundamental fact about stress tensors is that, at any point in a rock mass, there necessarily exists three mutually perpendicular directions that form a Cartesian coordinate system in which the shear stresses are all equal to zero [11]. In this coordinate system, the only non-zero components of the stress tensor are the three diagonal components, which are known as the *principal normal stresses*.

In the interior of a rock mass, i.e. at a location that is not at an open surface such as a borehole or tunnel, the three normal stresses are always compressive and are referred to as the maximum, intermediate and minimum normal stresses. Typically, and almost invariably at depth, one of the principal normal stresses will be oriented in the vertical direction and will be solely due to the weight of the overlying rock. The other two principal normal stresses therefore act in two mutually perpendicular horizontal directions. If a rock is porous, a pore fluid pressure will act throughout the rock, playing to some extent the role of a tensile normal stress acting equally in all three directions. The deformation of a rock is due to the totality of these stresses, as explained in the following section.

### 2.3. Elastic properties

The mechanical properties of subsurface rocks are integral to understanding rock behaviour in response to geological and engineered processes. A recent review of geomechanical studies of caprocks in depleted hydrocarbon reservoirs used for pilot tests of subsurface carbon capture and storage [22] noted the scarcity of geomechanical data for caprocks in the context of carbon sequestration, underscoring the necessity for site characterization in order to be able to accurately predict and quantify leakage rates. The lack of such data, added to the general complexity of subsurface geomechanical behaviour, poses significant challenges in the efficient and sustainable utilization of subsurface resources. Therefore, a diverse mixture of data sources is essential to effectively navigate these uncertainties.

#### 2.3.1. Young’s modulus

The most basic and important mechanical properties are the *elastic moduli*, which quantify the ratio of stress to strain, and which consequently are crucial in understanding and modelling the mechanical deformation of rocks [11]. Young’s modulus, often referred to in the non-English-language literature simply as ‘the elastic modulus’, and usually denoted by *E*, is defined as the ratio of stress to strain under conditions of uniaxial stress. Since stress has units of Pascals and strain is dimensionless, Young’s modulus also has units of Pascals. Young’s modulus of a rock varies with its mineralogy, its location, its degree of weathering and its burial history. The numerical value of Young’s modulus depends on its mineralogy, its porosity and the microstructure of its pore space [23,24]. These numerical values can range from approximately 10 GPa for softer rocks such as some shales or sandstones to approximately 100 GPa for stiffer rocks such as gabbros or dolomites [25]. Although typically thought of as a ‘constant’ for a given rock, and usually treated as such in both numerical and analytical modelling, Young’s modulus generally increases with the level of applied stress and also exhibits hysteresis during loading and unloading cycles [26,27].

Young’s modulus and its variation during deformation can be measured using a conventional uniaxial or triaxial loading apparatus, as well as with more advanced cyclic loading methods that can capture the variation of the elastic modulus during dynamic loading [28,29]. More recently, nano-indentation has emerged as a convenient tool to evaluate the multi-scale mechanical properties of brittle rocks, capable of capturing deformation behaviour at the small scale, and its spatial variation, based on surface measurements [30,31]. Nano-indentation has been utilized to estimate the localized mechanical properties of individual grains of homogeneous and heterogeneous rocks such as shale, coal, limestone, marble, sandstone and claystone [32], including Young’s modulus but also measuring hardness, fracture toughness, time-dependent creep and tensile strength.

The alteration of the elastic modulus during stress changes is essentially due to the evolution of micro-scale damage in the form of fracturing or pore collapse. Sample damage, even in rocks that appear visually intact, which may reflect *in situ* conditions or may be the result of damage due to coring and subsequent core handling, can therefore substantially reduce Young’s modulus, thus highlighting the importance of sample selection and preparation when aiming to measure these properties accurately. *In situ* methods to measure Young’s modulus at depth rely on expensive experimental set-ups such as the plate loading or jacking test, and the use of pressure chambers at depth, and have now largely been replaced by a combination of laboratory tests and empirical formulations that rely on the classification of rock samples according to their strength and quality [33].

Chemical reactions between the pore fluid and the rock can also cause softening phenomena, resulting in a decrease in the elastic modulus. For mudstones, this effect may be quite substantial, in some cases causing more than 30% reduction of elasticity modulus for 1% water content for swelling mudstone samples [34]. Song *et al.* [35] considered the effect of microscopic rock structure on the elastic modulus of sandstones using micro-computed tomography imaging and reported a correlation between tortuosity of the rock skeleton and the elastic properties of the sandstone. Micromechanics-based constitutive models can be used to predict the elastoplastic behaviour, time-dependent deformation and induced damage of clay-rich rocks by accounting for porosity, mineral inclusions, structural anisotropy and water sensitivity [36]. The interplay between these properties has also been recently investigated using the Mori–Tanaka homogenization scheme to model constitutive behaviour based on the anisotropic microstructure of the rock, taking into account elastoplastic deformation, time-dependent behaviour as simulated using the finite-element method and induced damage through a two-step homogenization process that integrates mineral compositions and porosity to determine the macroscopic elastic tensor and plastic yield criterion, while also considering interfacial de-bonding between the matrix and inclusions to capture rock damage in clay-rich rocks [37].

Young’s modulus will also be affected by extreme changes in temperature and generally decreases as the temperature increases. Despite this known fact, measurement of elastic moduli at elevated temperatures is not typically part of routine rock property measurements. Shen *et al*. [38] investigated the effects of water and supercritical CO_2_ injection on the mechanical properties of granite at high temperatures, highlighting the significant influence of confining pressure, pore fluid and temperature on the stiffness and strength of granite specimens. However, recent nano-indentation measurements performed for the thermo-mechanical characterization of shales over a temperature range of 25 to 300°C did not find a significant effect on the elastic modulus [39]. It has been found that in the context of hydrogen injection in a depleted gas field, a temperature difference of 20°C between the fluid and the reservoir can lead to additional deformation of reservoir and caprock, with thermo-poroelastic induced wellbore and reservoir damage resulting from local reductions in fracture pressures [40].

Uncertainties in the numerical values of rock property parameters pose significant challenges in the efficient and sustainable utilization of subsurface resources. These uncertainties arise from the complex and heterogeneous nature of subsurface formations. Ziegler [41] recently numerically investigated the effect of the variation in rock properties and showed how these lead to uncertainties in the predicted stress state, with Young’s modulus variability having the strongest influence.

#### 2.3.2. Bulk modulus

The bulk modulus of the rock quantifies its resistance to ‘hydrostatic’ compression, i.e. uniform compression in all three mutually perpendicular directions. It is numerically defined as the ratio of the hydrostatic pressure to the volumetric strain and is usually denoted by *K*. Similar to Young’s modulus, *K* has units of Pascals. Although the numerical values of the bulk modulus (very) roughly correlate with Young’s modulus, in the sense that ‘a rock having a large Young’s modulus will have a large bulk modulus’, in general the correlation is weak, and these two moduli must be independently measured for a given rock. Due to the rough correlation mentioned above, numerical values of bulk modulus tend to be 2−3 times less than the values of Young’s modulus [25].

Aside from controlling the deformation of a rock mass due to changes in hydrostatic stress or pore fluid pressure, the bulk modulus directly influences the speed of propagation of compressional ‘P-waves’, which are used in seismic prospecting to identify subsurface geological structures. The relationship between dynamic and static bulk modulus has been investigated, revealing the influence of petrographic parameters and porosity on the deformation behaviour of rocks, with nonlinear logarithmic and power correlations for igneous rocks, linear correlation for linear rocks, and nonlinear logarithmic and power correlation for metamorphic rocks [42].

The bulk modulus of a sedimentary rock depends strongly on the pore structure, with the pores usually modelled as oblate spheroids [23,24]. Pores cause the bulk modulus to decrease below that of the rock minerals, with this lowering effect being a minimum for nearly spherical pores (aspect ratio near 1), and being the greatest for thin crack-like pores (aspect ratio near 0). Relationships between bulk modulus in shale reservoirs and various petrographical parameters have been investigated, revealing a linear correlation between the average formation bulk modulus and the volume fraction of total organic carbon [43]. Semi-empirical rock physics models can be used to predict the macroscopic dynamic bulk modulus of heterogeneous rocks by taking as an input the compositional volume fractions of solid constituents, but still requiring empirical coefficients [44], indicating the challenges in predicting the change in the bulk modulus of the rock due to variations in solid properties.

#### 2.3.3. Shear modulus

The shear modulus of a rock, usually denoted by *G*, but occasionally by *μ*, quantifies its resistance to shear deformation—i.e. angular distortion, in contrast to the elongation or shortening along a given axis that is quantified by Young’s modulus. Since shear stresses exist whenever the normal stresses in two perpendicular directions are unequal, shear stresses are actually ubiquitous in the subsurface and consequently play an important role in controlling rock deformation.

Similar to Young’s modulus and the bulk modulus, the shear modulus of a rock has units of Pascals, and its numerical value depends on its mineral composition, pore structure, environmental conditions and loading history, and is also influenced by various factors such as moisture content, pore fluid and serpentinization [45]. The influence of pore water on the shear modulus of rocks varies significantly depending on factors such as rock type, porosity and mineral composition. Rocks such as silty clay, sandstone and shale typically experience a significant reduction in shear modulus due to the presence of water in the pore space, whereas denser rocks such as granite, basalt and quartzite exhibit a less pronounced effect, owing to their lower porosity and limited water retention capacity within their denser solid matrix [46]. Numerical values of the shear modulus are approximately half of those of Young’s modulus [25].

The propensity of pore water to significantly reduce the shear moduli of rocks has been investigated for limestones [47,48], sedimentary rocks [49] and carbonates [50,51]. For clay-rich rocks, shear softening can be caused by chemical reactions between the solid frame and the pore fluid in the rock, with the variation attributed to factors such as fluid–solid interaction, clay degradation and viscous coupling [52]. In addition, as in clay-rich rocks, shear softening is also driven by pore-scale interfacial phenomena effects, dependent on surface tension between immiscible fluids, rock wettability, aperture distribution, compressibility and porosity of microcracks, elastic properties of rock mineral, fluid saturation, effective stress and wave amplitude [53].

#### 2.3.4. Poisson’s ratio

The Poisson’s ratio, usually denoted by *ν*, although occasionally by *σ*, is a dimensionless parameter that quantifies the ratio of transverse expansion to axial shortening when a material is compressed uniaxially. Poisson’s ratio is actually not an independent parameter, as it is directly expressible in terms of the ratio of any pair of Young’s modulus, bulk modulus and shear modulus [11]. Nevertheless, Poisson’s ratio conveys subtle information about a rock’s microstructure [54] and its fluid saturation state [55], and is therefore of great interest in its own right. Poisson’s ratio of a dry rock must lie between 0 and 0.5; it increases towards the theoretical upper limit of 0.5 when the rock is fluid saturated. In particular, small values of *ν* generally indicate the presence of large numbers of dry or gas-filled open cracks, whereas large values of *ν* are often indicative of liquid-saturated pores and cracks [56]. The Poisson ratio also uniquely controls the ratio of the speed of compressional waves to the speed of shear waves [24] and is therefore very useful in seismic exploration and monitoring.

#### 2.3.5. Elastic anisotropy

The discussion in the previous sections has focused on ‘isotropic’ rocks, for which the elastic properties are independent of direction. But many sedimentary rocks, particularly shales, exhibit anisotropic mechanical properties, which are different in, for example, the horizontal and vertical directions. In shales, this anisotropy is due to factors such as partial alignment of anisotropic clay particles, kerogen inclusions, microcracks, low-aspect-ratio pores and layering [57]. Although there are many types of elastic anisotropy, each having its own unique number of distinct elastic moduli, rocks such as shales generally have different Young’s moduli in the vertical direction than in the horizontal direction and different Poisson ratios in a horizontal plane than in a vertical plane [58].

Recent advances in the application of the digital volume correlation method to analyse X-ray computed tomography images have enabled the investigation of the anisotropic elastic moduli of rocks such as shales at elevated temperatures, under triaxial loading conditions [59]. On a reservoir or repository scale, the elastic properties are often influenced by the presence of *in situ* fractures, and the macroscopic properties will depend on the sizes and orientations of these fractures [60].

### 2.4. Faults and fractures

When rocks are formed, whether through igneous or sedimentary processes, they initially do not contain any fractures or other open discontinuities. But cracks can form in rocks due the action of stresses, temperature changes or pore pressures. If these cracks are longer than a few centimetres in length, they are usually referred to as *fractures*. If the two opposing faces of a rock fracture show evidence of previous tangential displacement relative to one another, this fracture is referred to as a *fault* [61,62]. Faults may have lengths ranging from tens of metres to many kilometres. Most subsurface rock formations contain many fractures and many are heavily faulted.

Fractures and faults may be mainly ‘open’, in which case they provide rapid conduits for fluid flow, or they may be partially (or in some cases, totally) filled with minerals [63] and therefore relatively impermeable. They are, in either case, typically much weaker than intact rock and are therefore liable to undergo additional shear displacement, due to changes in stress or pore pressure. Compressive normal stresses tend to ‘prevent’ a fault from slipping, whereas shear stresses tend to promote slip. Since pore fluid pressures act as ‘extensional’ normal stresses, pore pressures also promote slip.

The simplest and most commonly used model for the initiation of slip on a fault is the Coulomb equation, *τ* ≥ *S_o_ + μ*(*σ-p*), where *τ* is the shear stress acting on the fault plane, *σ* is the normal stress acting on the fault, *p* is the pore fluid pressure, *μ* is a fault property known as the coefficient of internal friction and *S_o_* is a fault property known as the cohesion [11]. The resultant shear displacement usually causes the permeability of the fracture to increase, in some cases substantially. It is clear from the Coulomb equation that fault slip can be initiated either by an increase in shear stress, a decrease in normal stress or an increase in pore fluid pressure.

As discussed in more detail in §4, faults and fractures are key to many subsurface green energy technologies. Since enhanced fluid pressures tend to cause tangential slip along a fault surface, and such slippage may lead to an earthquake, the possibility of induced seismic activity exists when fluids are injected during the subsurface storage of CO_2_ or hydrogen. In other subsurface green energy technologies, such as engineered geothermal systems (EGSs), the creation of new fractures is desired, as they increase the permeability of the subsurface rocks and create more surface area for heat transfer.

### 2.5. Strength properties

Tensile and compressive strength are the parameters that quantify the maximum tensile and compressive stress, respectively, that a rock can withstand before it fails. The process of ‘failure’ may in some cases refer to the creation of a through-going fracture plane and in other cases may refer to other, more localized changes in microstructure (cracking, pore collapse, etc.) that cause the rock to be unable to withstand a higher stress. Brittle rocks exhibit different behaviour under tension and compression, and in particular, its tensile strength is typically approximately 10 times less than its compressive strength [11,46]. The use of different testing methods, such as direct tension, Brazilian splitting and three-point bending, has provided insights into the tensile strength and damage evolution process of various rocks [64]. Numerical and experimental studies have shown that tensile fractures in Brazilian splitting typically initiate as the initiation of pre-existing micro-flaws and micro-fractures that reactivate, propagate and coalesce in response to tension [65,66].

Tensile and compressive strength are essentially ‘macroscopic’ properties that make no explicit reference to microscopic processes such as might occur at the tips of cracks, for example. But on a microscopic scale, failure often occurs by the growth of pre-existing cracks. The propensity of a crack to grow is quantified by the *stress intensity factor* (SIF), which depends on the size of the crack, the elastic moduli of the rock and the stresses that exist in the neighbourhood of the crack [11,62]. Cracks are assumed to grow when the SIF reaches a critical value, which is a property of each rock, known as the *critical SIF*. There are in fact three different critical SIFs, denoted as {*K*_Ic_, *K*_IIc_, *K*_IIIc_}, corresponding to three different modes of crack growth. In mode I, the crack faces move apart as the crack extends in its plane; in mode II, the crack faces slide over each other in the plane of the crack in a direction normal to the edge of the crack; and in mode III, the two crack surfaces slide relative to one another in directions parallel to the edge of the crack [11].

Rock anisotropy has a strong influence on rock strength [67]. As an example, experimental tests on Longmaxi shale samples showed that bedding planes have significantly lower tensile strength, cohesion, internal friction angle and mode-I fracture toughness compared with the rock matrix, emphasizing their role as planes of weakness and their critical impact on fracture geometry and shale gas development [68]. To study the effects of anisotropy and heterogeneity on shale failure modes and tensile strength, Brazilian splitting tests were performed on shale samples at various bedding and loading angles. Results showed different crack growth patterns depending on the loading angle, with tensile strength increasing as the angle between the maximum normal stress and the bedding planes increased from 0° to 90°, with water significantly reducing tensile strength [69].

Xi *et al*. [70] investigated the dynamic tensile properties of granite subjected to various thermal treatments and cooling methods (natural, water and liquid nitrogen) using ultrasonic testing and split Hopkinson pressure bar tests. Their results showed that as the thermal treatment temperature increased, P-wave velocity, dynamic tensile strength and absorbed energy each decreased, with liquid nitrogen cooling having the most significant effect on thermal shock damage, followed by water and natural cooling. Recently, Jin *et al.* [68] heated granite from 25 to 1000°C and performed triaxial compression tests on specimens cooled by water, revealing that differential thermal expansion coefficients of mineral particles are the main drivers of thermal cracks, with a formation threshold between 500 and 550°C, showing elastic-plastic behaviour at 1000°C. These findings, which are furthermore confirmed by numerical models, provide further evidence that the strength of granite decreases substantially with increased thermal exposure, due to changes occurring at the microscale. The physical changes involved include changes in mass and volume, and loss of internal moisture, whereas chemical changes include variations in mineral composition and crystalline phase transitions. Chemical changes of the rock minerals can also occur in the absence of large temperature changes. For example, a study focused on the influence of acid-base corrosion of the toughness of marble showed a decrease of the toughness by nearly a factor of two, and an increase of crack growth velocity when subjected to an acidic solution with pH of 3, due to the weakening caused by the dissolution of calcite, dolomite and mica minerals in the rock matrix [71].

The property of *toughness* is a measure of a rock’s ability to absorb energy and deform before fracturing. The toughness of a rock is influenced by its mineral composition, grain size and the presence of microcracks or flaws. Toughness influences resilience to fracturing and deformation and is critical for assessing the suitability of a rock for storing pressurized fluids, as it is instrumental in predicting fracture growth. The measurement of rock toughness has been a subject of extensive research. The International Society for Rock Mechanics has developed standard methods for determining the static fracture toughness of rock [72]. New methods for determining dynamic fracture toughness of rocks, such as the internal central single-cracked disc specimen for blast loading, have been proposed, emphasizing the continuous development of techniques for rock toughness measurement [73].

Studies have shown that fracture toughness of rocks can be influenced by factors such as relative humidity, temperature, high-temperature treatment and chemical corrosion [74]. The fracture toughness of granite and gabbro has been observed to decrease with increasing temperature, attributed to the density and length of microcracks increasing due to thermal stress, resulting in accelerated macrocrack propagation [75]. A study of sandstone toughness measurements revealed that fracture toughness decreases with increasing temperature, whereas the fractal dimension of the fracture profiles increases. Temperature also affects the fracture mode, with transgranular failure occurring at lower temperatures (20−400°C) and intergranular failure at higher temperatures (400−700°C). The weakening mechanisms involve thermal dehydration between 100 and 400°C, and thermal cracking that occurs due to the α–β phase transition of quartz at 573°C [76].

Numerous recent studies have advanced the understanding of temperature effects on rock properties, from detailed numerical simulations of rock strength to theoretical models predicting fracture toughness and tensile strength. Recent theoretical models include that of Qiu *et al*. [77], who developed a temperature-damage-dependent model of mode I fracture toughness. Their theoretical framework, validated by experiments, accurately predicted fracture toughness at various temperatures. Zhao *et al*. [78] proposed a model based on the force-heat equivalence energy density principle, to predict tensile fracture strength across temperatures. Their models account for phase transitions and thermal damage, offering a rapid and practical evaluation method for rock strength.

Saksala [79] developed a numerical method using embedded discontinuity finite elements to predict the tensile strength of granitic rock under temperature variations. By incorporating the temperature-dependent thermal expansion of quartz, and using a staggered explicit time-stepping approach, that study effectively matched experimental data on thermal cracking and strength degradation. Building on that work, Saksala [80] extended the approach to include both tensile and compressive strength as well as stiffness. Utilizing a damage-viscoplasticity model based on the Drucker–Prager criterion, this study incorporated rock heterogeneity and demonstrated realistic predictions of temperature-induced weakening and failure modes. Pressacco *et al*. [81] examined the effects of thermal pre-treatments, including conventional heating and microwave irradiation, on granite-like rock. They used a damage-viscoplasticity model and finite-element-based simulations, to compare energy consumption and induced damage. Their findings highlighted how thermal-based pre-treatment methods can be used to enhance rock fracturing efficiency by reducing strength.

### 2.6. Transport properties

Many of the processes that occur in conjunction with subsurface green energy technologies involve not only mechanical deformation but also the flow and transport of fluids and heat. Although the focus of this review is on geo*mechanical* processes, many of these processes involve coupling between mechanical deformation, fluid flow and heat transport. Hence, a brief introduction to transport processes and their related rock properties is pertinent.

#### 2.6.1. Permeability and Darcy’s Law

The flow of a fluid through a porous rock is governed by a law known as Darcy’s Law [82]. This law essentially states that the volumetric flow-rate of a fluid through a porous rock is proportional to the fluid pressure gradient, inversely proportional to the viscosity of the fluid and proportional to a rock property known as the *permeability*, and is almost invariably denoted by *k*. High permeability is usually desired for activities such as hydrogen or CO_2_ storage, since high permeabilities will allow fluid to be injected under lower injection pressures, and to rapidly penetrate from the injection wells into the entire reservoir. Rock formations having low permeabilities, such as granites or some mudrocks, are preferred in situations where fluid transport is to be avoided, such as nuclear waste disposal [83].

The permeability of a rock depends on its porosity, and also on the topology (interconnectedness) of the pore space. But in contrast to most rock properties, which have no intrinsic dependence on the *size* of the pores, permeability generally depends on the square of the pore diameter, and in fact has units of m^2^. Due to this strong dependence on pore size, the permeability of different rocks varies by many orders of magnitude, ranging from 10^−12^ m^2^ for some sandstones, down to as low as 10^−19^ m^2^ for some shales [84] or granites [85]. Although often treated as a constant parameter in modelling exercises, permeability generally decreases with increasing stress, as the small microcracks that provide pathways for fluid flow close up [86].

On a scale larger than the rock cores that are typically used in laboratory measurements of permeability, the permeability of a rock mass on a reservoir or repository scale is in many cases controlled by a network of fractures. The ‘permeability’ of a single fracture is proportional to the square of its aperture (approximately the normal distance between the opposing rock walls), and the macroscopic permeability of a fractured rock mass depends in a complex way on the apertures of the fractures, the spacing between fractures, and interconnectedness of the fractures [63]. In some rock masses, such as the crystalline rock that will host the geological disposal facility for nuclear waste at Olkiluoto in Finland, the intrinsic permeability is so low, perhaps as low as 10^−20^ m^2^, that the rock matrix can effectively be considered to be impermeable, with fractures providing the only potential paths for the flow of fluids and the transport of radionuclides [87,88].

#### 2.6.2. Thermal conductivity and Fourier’s Law

The flow of heat through a rock, whether porous, fractured or intact, is governed by a law known as Fourier’s Law [89]. This law is mathematically analogous to Darcy’s Law, in this case with the heat flux being proportional to the *temperature* gradient, and proportional to a rock property known as the *thermal conductivity.* Thermal conductivity has units of W m^−1^ K^−1^ and is usually denoted by *k*, although in situations involving both heat flow and fluid flow, it is often denoted by *λ*, to avoid confusion. The numerical value of the thermal conductivity of a rock depends on its mineral composition [90]. For porous rocks that are saturated by fluids, the thermal conductivity will also depend on the thermal conductivity of the pore fluid, the porosity and the pore shape [91].

In contrast to permeability, which varies by almost 10 orders of magnitude between different rock types, the thermal conductivity of different rocks varies only within the relatively narrow range of approximately 1−10 W m^−1^ K^−1^ [90]. Dry porous rocks generally have lower thermal conductivities than liquid-saturated rocks [91]. Thermal conductivity tends to vary more strongly with temperature than do most other physical properties, although the variation is not always smooth, and can in some cases be non-monotonic [92].

In transient subsurface processes, the key thermal property is actually not the thermal conductivity, but rather the *thermal diffusivity*, which governs the rate at which temperature pulses propagate through the rock. The thermal diffusivity, *D*, is defined as the thermal conductivity divided by the heat capacity per unit volume and has units of m^2^ s^−1^. Roughly speaking, the front of a temperature pulse will propagate a distance of (*Dt*)^1/2^ over an elapsed time of *t*. The thermal diffusivity is a key parameter in EGSs, where high thermal diffusivities are obviously advantageous. In a geological disposal facility for nuclear waste, the thermal diffusivity controls the rate at which the high temperatures generated by the radioactive decay of the waste will propagate away from the waste package deposition holes [93]. However, due to the relatively narrow range of variation of both thermal conductivity and heat capacity, the thermal diffusivities of different rocks vary over a very small range and almost always equal 10^−6^ m^2^ s^−1^, plus or minus a multiplicative factor of 2 [94].

## 3. Green energy technologies and subsurface geomechanics

### 3.1. Carbon sequestration

It is now widely agreed that the Earth is undergoing an unprecedented period of rapid climate change, driven primarily by the increased presence in the atmosphere of carbon dioxide and other ‘greenhouse gases’ such as methane, ozone, nitrous oxide and chlorofluorocarbons [1]. These gases tend to absorb some of the radiation emitted by the Earth, which would otherwise radiate into space. The effect is to cause the mean temperature of the Earth to increase. A large fraction of all greenhouse gases emitted into the atmosphere are by-products of the burning of fossil fuels, such as oil, natural gas and coal, for the purpose of generating electricity, heating residential or commercial spaces and powering automobiles, planes and other transport vehicles. Global annual CO_2_ emissions from fossil fuels and various industries, estimated at 37.79 billion tonnes, continue to soar and are not expected to have peaked yet [95]. One method of slowing down or controlling global warming due to greenhouse gases is to capture the carbon dioxide, either at the source of its generation (i.e. at coal-fired power stations) or by stripping it from the atmosphere by physico-chemical means and then sequestering it in the subsurface [96,97].

**Figure 1.**
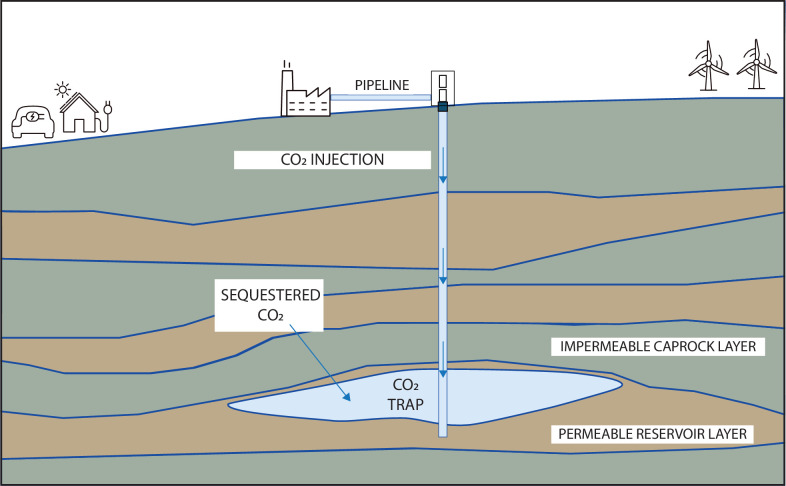
Geological carbon sequestration is aimed at permanently storing large volumes of carbon dioxide in the subsurface. Over time, the CO_2_ will mineralize, becoming immobile. On shorter timescales, the process will rely on the injected CO_2_ being trapped by the overlying impermeable caprocks.

The most promising locations for sequestering this carbon dioxide in the subsurface are either saline aquifers, which due to their high salt content cannot otherwise be used as sources of drinking water, or depleted hydrocarbon reservoirs (figure 1). Depleted hydrocarbon reservoirs have the obvious advantage of already having proven, by virtue of having stored liquid or gaseous hydrocarbons for millions of years, that they are capable of storing non-aqueous fluids for extended periods, without appreciable leakage of these fluids back to the surface. Over longer timescales (thousands of years), the carbonate ions that have dissolved in the brine will react with minerals and precipitate as solid carbonates, which will essentially be ‘locked’ into the rock formation [98,99]. However, for engineering timescales of years and decades, which are most relevant and crucial to the aim of mitigating global warming due to greenhouse gases, the most important mechanism for carbon sequestration will be the hydrodynamic trapping of the carbon dioxide under stratigraphic or structural traps that are overlain by impermeable caprocks. Common types of traps are, for example, locations where the sedimentary rock layers have formed a dome-like shape, allowing the buoyant hydrocarbons to rise into the dome, with further migration blocked by the presence of an impermeable caprock.

In order to sequester the carbon dioxide most efficiently, it would be desirable to inject it into the subsurface at a high density, so as to maximize the mass of carbon dioxide that can be stored in a given pore volume of rock. Fluid density generally increases with pressure, but the maximum injection pressure is constrained to be sufficiently low so as not to cause fracturing of the overlying caprock [20]. Consequently, the CO_2_ will most likely be injected as a supercritical fluid, at densities in the range of 266–733 kg m^−3^ [98], which are much less than the densities of brine, which, depending on the salinity, will range from approximately 1000 to 1200 kg m^−3^ [98]. Hence, the CO_2_ will be the less dense fluid and will tend to migrate either up-dip or into domes of depleted hydrocarbon reservoirs.

When CO_2_ is injected into a saline aquifer, it will by necessity displace some of the brine, creating a situation of two-phase flow. Some of the CO_2_ will dissolve into the brine, in which case this flow process should be treated as one of miscible flow [100]. The numerical simulation of the injection and migration of CO_2_ under two-phase conditions, with or without considering miscibility, has been the subject of hundreds of simulation studies over the past two decades [101,102] and several review articles [103]. As the focus of the present review is on geomechanical issues, purely hydrodynamic issues will not be emphasized. However, the important geomechanical implications of CO_2_ injection can only be properly modelled, whether numerically or analytically, on the basis of fully coupled hydro-mechanical models.

From the point of view of geomechanics, there are several issues that must be addressed in order to design a safe carbon sequestration project that will isolate the CO_2_ from the atmosphere for extended periods of time and which will not cause any associated disturbances that might make the project socially unacceptable. One mechanism by which the CO_2_ could potentially escape to the surface is if the caprock is breached, and a fracture, or network of fractures, is created that forms a hydraulically conductive path from the sequestration reservoir to the more permeable rock layers near the surface [104]. Even if the CO_2_ does not re-enter the atmosphere, it may pollute subsurface drinking water sources. Another possible route for the CO_2_ to escape is for a pre-existing fault to be sheared, again possibly creating a permeable flow path from the reservoir to the near surface [105,106]. A third possible escape route for the CO_2_ would be flow upwards through an abandoned but imperfectly sealed oil or gas well [107].

Aside from the risk of leakage of the CO_2_, there is also a risk that the injection of the CO_2_ will cause unwanted effects such as induced seismic events. It is well known that injection of fluids into the subsurface under high pressure can cause earthquakes [108–110]; this phenomenon is referred to as ‘induced seismicity’. These earthquakes can cause property damage and loss of life, so the possibility that CO_2_ may cause earthquakes in the vicinity of the sequestration reservoir will be detrimental to the social acceptance of subsurface sequestration at a given site. These geomechanical issues associated with subsurface CO_2_ sequestration will be discussed in §4.

### 3.2. Geothermal energy

The temperature at the centre of the Earth is approximately 5200°C, whereas the mean surface temperature of the Earth is approximately 15°C. Hence, there exists a temperature gradient within the Earth that causes heat to flow upwards towards the surface [111]. The thermal energy content of the Earth can, in principle, be used for electricity generation, or space and water heating, which together constitute approximately half of human energy consumption of approximately 20 TW [112]. However, the spatial density of the geothermal heat flux is low, averaging only 0.065 W m^−2^ over the Earth’s land surface [111]. If *all* of the heat flux that reaches the Earth’s land surface of 1.48 × 10^14^ m^2^ could be captured and used for electricity generation or residential/commercial heating, this would still fall short of the current world energy usage rate for electricity and heating, which is approximately 10 TW [112]. However, there is a large amount of geothermal energy already *stored* in the upper few kilometres of the Earth’s crust, which can serve as a viable, if not actually renewable, alternative to fossil fuels.

Consequently, the use of geothermal energy for either electricity generation or industrial/residential heating has been pursued for over a century, and geothermal energy is widely viewed as potentially making a substantial contribution to future energy requirements. Geothermal energy can broadly be broken down into three modes: (i) extraction of high-enthalpy fluids from the subsurface, to be used to generate electricity via steam turbines, (ii) extraction of fluids from the subsurface to be used directly to provide residential or commercial heating, and (iii) ‘EGSs’ in which cold water is injected into the subsurface, absorbs heat from the relatively hotter rock, and is then pumped back up to the surface to be used for electricity generation or heating.

There are a small number of ‘vapour-dominated’ geothermal reservoirs worldwide that contain high-enthalpy fluids (i.e. steam) that can be extracted to produce appreciable amounts of electricity by running the fluid through a steam turbine [113]. In decreasing order of electrical power production, these include The Geysers in northern California (USA), Cerro Prieto in Baja California (Mexico), and Larderello in Tuscany (Italy). Total electricity generation from vapour-dominated geothermal reservoirs currently accounts for approximately 0.1% of total worldwide electricity generation, and new such fields of appreciable size are not being discovered. The geomechanical issues that accompany electricity generation from these geothermal reservoirs are similar to those related to other energy technologies that involve subsurface extraction of fluids, such as land subsidence [114,115]. Often, fluids are injected into the reservoir to maintain pressures [116], and this may lead to unwanted induced seismicity [117,118].

**Figure 2:**
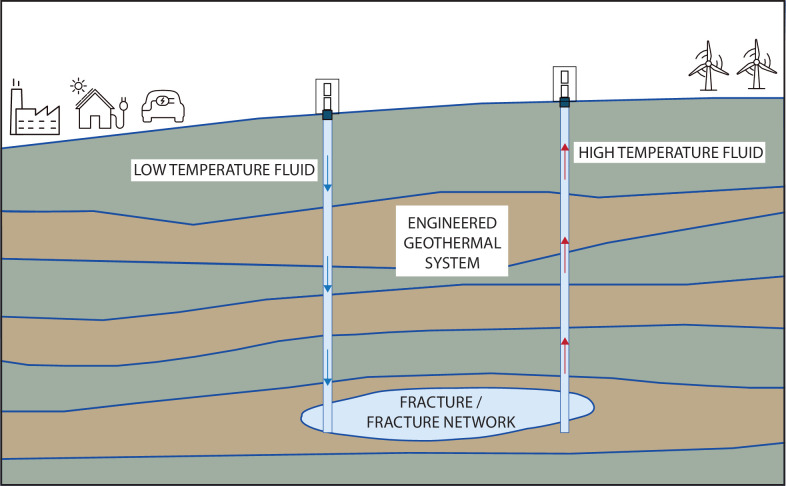
EGSs can mine heat from the subsurface. At high enough temperatures, this heat can be utilized to produce electricity; otherwise, the extracted hot water can be used for space heating. EGSs depend on a permeable region between the low-temperature injection well and the high-temperature extraction well, often created by inducing fractures to connect the two wells. Understanding the coupled thermo-hydro-mechanical behaviour of the subsurface system is crucial to creating a successful EGS.

The most promising technology for utilizing geothermal energy at a much greater scale than at present is to inject cold water through a borehole, allow the hot rock to heat the water, and then extract the water from a (typically different) borehole, thereby treating the hot subsurface rock as a large ‘heat exchanger’. The heated water can then be used to generate electricity, or simply to provide residential or commercial heating. These so-called ‘EGSs’ have the advantage of not requiring a subsurface rock with large porosity and/or permeability, and can therefore be sited in essentially any location (figure 2). However, rocks have very low thermal diffusivities [111], and so heat transfer by conduction through the rock generally occurs at too slow a rate for the process to be technically viable. Hence, a typical configuration of an EGS is to have two parallel boreholes, one acting as an injector and the other as a producer, and to connect them by creating one or more hydraulic fractures between the injector and the producer. The injected water then flows down the injector well, through the fracture, to the production well. The fracture allows a large surface area of contact between the flowing fluid and the rock, thereby allowing a large overall heat transfer rate, despite the low thermal diffusivity of the rock matrix [19].

Geomechanics plays a key role in EGSs. Aside from the aforementioned issue of potential induced seismicity that exists whenever fluids are injected into the subsurface, EGS projects are crucially dependent on hydraulic fracturing, the modelling of which involves complex coupled thermo-hydro-mechanical processes [12,119,120].

### 3.3. Hydrogen storage

The amount of electricity that is generated by renewable energy sources such as wind or solar energy is increasing steadily, both in relative and absolute terms. An obvious weakness of both of these energy sources is their intermittent nature. As electricity *per se* is not easily stored in large quantities, it has been suggested that during periods of excess electrical production, electricity produced from wind or solar energy could be used to produce ‘green hydrogen’, through an electrolysis process that produces hydrogen from water, creating no CO_2_ or other harmful waste products [121]. This hydrogen could then be stored and utilized to produce electricity during the periods when solar and/or wind energy is relatively unavailable. If successful, this scheme would reduce reliance on fossil fuels as ‘emergency seasonal fuels’ [122].

**Figure 3.**
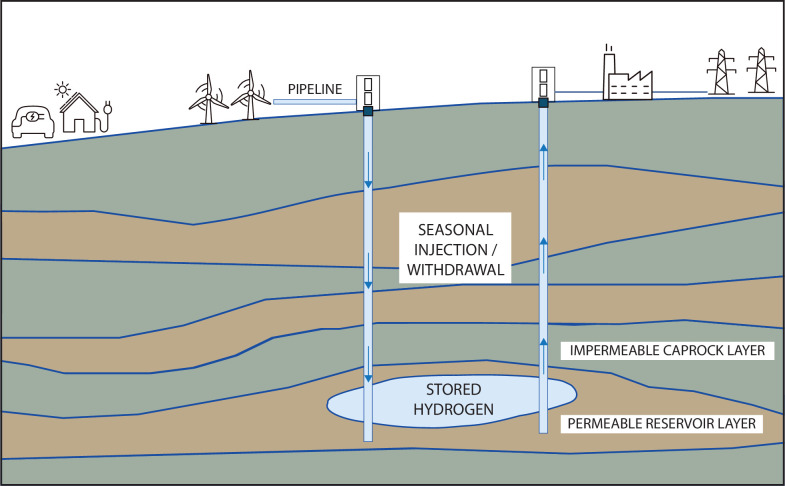
‘Green’ hydrogen, produced from green energy sources, can be injected into the subsurface and then extracted during periods of higher electricity demand and used to generate electricity. This technology could contribute to the optimization of sustainable energy consumption and help to balance power grid supply over longer periods.

As large volumes of hydrogen would be needed for this process to have a non-trivial effect on reducing greenhouse gas emissions, surface-based storage of this hydrogen in man-made containers is not feasible at the required scale. Hence, it is envisioned that the hydrogen would be stored in the subsurface (figure 3), either in porous rock formations such as depleted hydrocarbon reservoirs or saline aquifers [123] or in large underground caverns created in, for example, salt rock [124–126].

If hydrogen is stored in porous subsurface rock formations, geomechanical issues will arise that are similar to those that are faced during CO_2_ sequestration: induced seismicity, surface subsidence and the mechanical integrity of the caprock [127,128]. Induced seismicity, which is likely to be caused by the stress state acting on an existing fault moving closer to the failure condition, may result either from an increase in the pore fluid pressure or from an increase in the shear stress [129,130], both of which may occur as a result of the cyclic injection/withdrawal of large volumes of hydrogen.

Withdrawal of fluid from the subsurface may cause a lowering of the ground surface, which is known as subsidence. Although generally not a threat to life, subsidence can cause property damage to buildings and other surface structures and is therefore a potentially unwanted side effect whenever fluids are extracted from the subsurface [131]. Another hazard that may accompany repeated injection and withdrawal of hydrogen from a subsurface reservoir is the possibility that the caprock will be mechanically breached, either by the creation of new fractures or the reactivation of existing faults [131], thereby allowing the gas to escape to the surface.

Another possible mode of subsurface hydrogen storage is to store it in large artificially created caverns in salt rock [132]. These caverns can be created by a process known as solution mining, in which water is injected into the salt formation, dissolving some of the salt rock [133]. The caverns thus created may have volumes as large as 10^6^ m^3^ and are approximately oval shaped, with their long axis oriented vertically. The main geomechanical hazards associated with storage of hydrogen in salt caverns are roof collapse and leakage to the surface through crack pathways [134].

### 3.4. Nuclear waste disposal

Currently, approximately 40% of energy-related emission of carbon dioxide into the atmosphere is due to coal-fired or gas-fired electrical power plants [135], which create carbon dioxide as a by-product of the combustion process. Power plants that generate electricity by combusting fossil fuels or biofuels account for approximately two-thirds of all electrical power generation worldwide [136] and approximately 44% in the UK [112]. Nuclear power plants, on the other hand, although posing their own set of environmental risks and challenges, generate essentially no greenhouse gases [136]. Consequently, replacing some sizable fraction of electricity that is currently generated by burning fossil fuels or biofuels, with electricity generated from nuclear fission, offers the possibility of substantially reducing greenhouse gas emissions.

**Figure 4.**
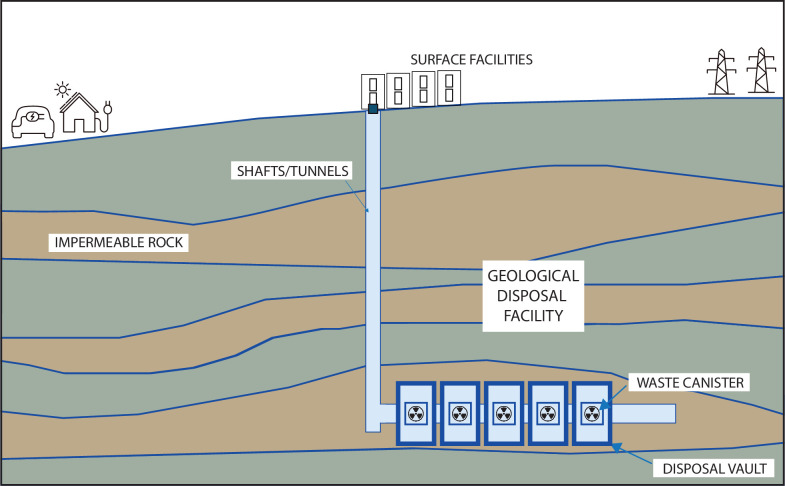
Subsurface nuclear waste disposal requires the construction of large underground geological disposal facilities for the safe, long-term containment of nuclear waste. Isolation of the waste in the GDF will require the waste to be encased in canisters, surrounded by the ‘engineered’ barrier, in turn surrounded by the ‘natural barrier’ of impermeable rock. Adapted from https://environmentagency.blog.gov.uk/2021/12/01/regulating-a-geological-disposal-facility-to-protect-the-environment/.

Generation of electricity by nuclear fission, although producing no greenhouse gas emissions, does produce substantial quantities of highly radioactive waste, most notably in the form of depleted fuel rods. This waste contains various radionuclides (radioactive isotopes of elements such as, for example, uranium or plutonium), each having its own half-life, some of which will remain radioactive for many thousands of years. Most countries that produce radioactive waste, including England and Wales, have decided that the best mechanism for the safe disposal of this waste will be to isolate it in specially engineered subsurface geological disposal facilities (figure 4), referred to as GDFs [137,138].

In the Swedish disposal concept, for example, the spent fuel rods will be entombed in large cylindrical copper-clad canisters, approximately 5 m long and slightly greater than 1 m in outer diameter [139], which will then be placed vertically in deposition boreholes that have been drilled below the floor of horizontal tunnels. The planned GDF in Sweden will be located in a granitic host rock, near Forsmark on the Baltic coast, at a depth of approximately 500 m. The space between the copper waste canisters and the borehole wall will be filled with a clay-like material called bentonite, which swells upon contact with subsurface brine, thereby providing both mechanical stability and a layer of protection against migration, to the biosphere, of any radionuclides that might leak out of the canister as it decays [12,140,141].

As the main goal of nuclear waste disposal is to prevent the radioactive waste from reaching the biosphere, geological disposal facilities will be located in rock formations that are intrinsically impermeable to fluid flow. For example, the Swedish and Finnish GDFs will be located in granitic rocks, at Forsmark and Olkiluoto, respectively. The intrinsic matrix permeability of such rocks is of the order of 10^–21^−10^–18^ m^2^ [142], although the primary paths of fluid transport will be through pre-existing fracture networks [87] that have higher macroscopic permeabilities. The planned GDF in Switzerland will be located in the Opalinus clay formation, with an intrinsic permeability of 10^–21^−10^–20^ m^2^ [143], and the GDF in France is planned to be located in a Callovo-Oxfordian claystone having an intrinsic permeability of approximately 10^–20^ m^2^ [144].

Nuclear Waste Services (NWS) have the responsibility to oversee the design and construction of a GDF for radioactive waste in England and Wales. This GDF will be constructed at a depth of between 200 and 1000 m, but the site of the GDF has yet to be chosen [145]. A volunteer siting process has been initiated, and currently, three ‘Community Partnerships’ have put themselves forward for consideration as the location of the future GDF. Broadly speaking, three types of potential host rocks have been identified: rock salt, lower strength sedimentary rock and higher strength rocks [138]. Each of these types of rock is widely distributed throughout England and Wales, and NWS consider that, in principle, each of these types may be suitable to host a GDF.

Ultimately, the performance of a geological disposal facility is determined by the ability of radionuclides to escape from the waste canisters and flow or diffuse through the pore space of the host rock and reach the biosphere. But since GDFs will be constructed in formations of very low intrinsic permeability, failure to contain the waste can only occur if geomechanical processes substantially alter the transport capability of the host rock. This may occur due to mechanical degradation of the rock in the immediate vicinity of the GDF due to damage caused by the excavation process [16,146]. As radioactive waste is required to be safely sequestered for tens of thousands of years, the changes in subsurface stresses due to future glaciation cycles, or other natural processes such as earthquakes, must also be considered when assessing the performance of a GDF [147].

## 4. Geomechanical challenges

### 4.1. Introduction

The geomechanical challenges associated with any subsurface activity encompass a spectrum of potential risks, including induced seismicity, reservoir compaction and subsidence, and the integrity of wellbores and caprock [148–152]. These challenges are particularly salient in the context of subsurface energy technologies such as carbon capture and storage, geothermal energy, nuclear waste disposal and underground hydrogen storage, where the viability of projects hinges on the secure containment of fluids within subsurface formations relevant for both environmental and operational safety.

### 4.2. Borehole stability and integrity

Subsurface energy technologies such as EGSs, carbon sequestration or hydrogen storage necessarily require the drilling of boreholes through which fluids can be injected into, or withdrawn from, the subsurface rock formation. Drilling a borehole (or tunnel, drift, etc.) in a rock will drastically alter the state of stress in the immediate vicinity of the borehole, generally moving the stress state at some locations around the borehole closer to satisfying the shear or tensile failure criteria, as compared with the original *in situ* stress state [11]. The first geomechanical issue to be dealt with in connection to the drilling of a borehole is therefore to avoid the collapse of the borehole through shear failure and to prevent unwanted hydraulic fracturing through tensile failure of the adjacent rock.

The starting point of the analysis required to ensure borehole stability is knowledge of the *in situ* stress state. The resultant state of stress around the borehole then depends, in a known way, on the *in situ* stresses and the orientation of the borehole [153]. If this state of stress is inserted into a failure criterion, potential failure can be investigated at all angular locations along the borehole, and failure can be avoided by appropriate choices of drilling mud density and borehole orientation [154–156].

Sanei *et al*. [157] investigated the impact of subsurface stresses on operational aspects such as wellbore stability and integrity, and caprock integrity. From an engineering perspective, Mason *et al*. [158] emphasized the importance of characterizing the chemical and mechanical changes in wellbore cement caused by CO_2_-rich brines. This understanding is crucial for predicting the enduring stability of wellbores in geologic CO_2_ settings, underscoring the urgency to address these coupled chemical–mechanical interactions. Allen *et al*. [159] presented a detailed analysis of the potential for borehole failure to occur due to injection into the Acorn CO_2_ Storage Site, offshore UK, and highlighted the importance of accurate estimation of the *in situ* stress state in the reservoir, prior to the drilling of the borehole.

Disposal of nuclear waste in a geological disposal facility does not generally require the drilling of boreholes for fluid injection/withdrawal, but does involve extensive drilling and/or blasting of tunnels and other underground excavations. The mechanical integrity of these underground excavations must also be ensured, and the analysis required is in many ways similar to that involved in analysing borehole stability. The geomechanics issues involved in ensuring the stability of these excavations have been reviewed by Zou and Cvetkovic [83]. Analysis of excavation damage and stability for the Forsmark (Sweden) and Olkiluoto (Finland) repository sites has been carried out by Rutqvist and Tsang [88], using the ROCMAS coupled thermo-hydro-mechanical simulator. Saceanu *et al*. [16] used the Imperial College Geomechanics Toolkit to model mechanical spalling around vertical deposition holes for waste canisters at the Forsmark GDF site, focusing on issues such as the minimum spacing required between adjacent deposition holes in order to avoid enhanced damage and fracturing.

### 4.3. Subsidence

**Figure 5.**
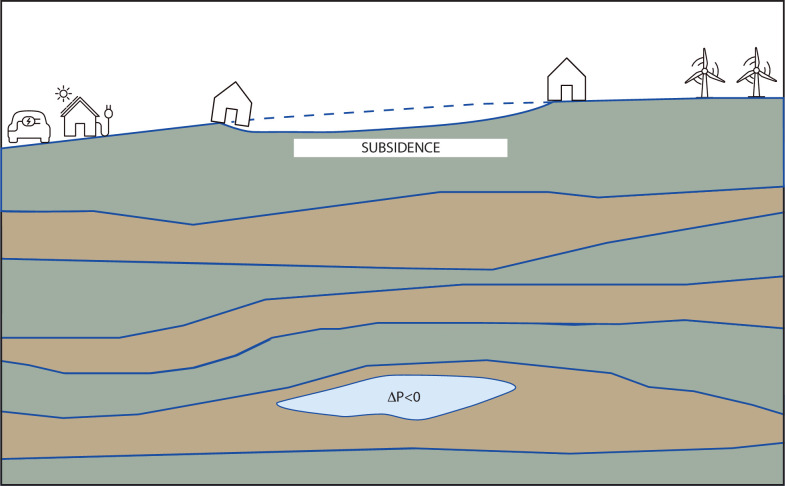
Extraction (or injection) of subsurface fluids will cause the pore fluid pressure to decrease (or increase), ultimately causing the ground surface to subside (or rise). This subsidence can cause physical damage to surface structures such a houses and other buildings. Surface deformations can be managed by proper rock characterization and operational constraints on fluid extraction/injection rates and volumes.

Withdrawal of fluids from a subsurface reservoir causes the ground surface above the reservoir to subside, essentially in the same manner that withdrawal of air from a balloon will cause the balloon to shrink (figure 5). This subsidence, typically of the order of millimetres or centimetres, is nevertheless capable of causing severe damage to surface structures such as houses, schools or hospitals [114]. Similarly, *injection* of fluids into the subsurface will cause *uplift* of the ground surface, which may also lead to damage of surface structures. Subsidence or and/or uplift is therefore liable to occur during carbon sequestration [104], seasonal gas storage and withdrawal [160] or enhanced geothermal energy production [161].

Much of the understanding of subsidence has been gained from studies that focused on oil and gas extraction, and this knowledge can be expected to carry over to other subsurface energy technologies that involve fluid injection and/or extraction. Smith and Knight [162] and van Thienen-Visser *et al*. [163] discussed the observable subsidence and seismicity induced by reservoir pore pressure depletion and highlighted the importance of understanding the long-term relationship between reservoir pore pressure depletion and rock compaction, in order to ensure safe and sustainable operations. Chang *et al*. [164] demonstrated the significance of post-depletion compaction in reservoir-surrounding shale, indicating the need to answer questions related to this phenomenon to explain the observed acceleration of subsidence that occurred *after* fluid depletion has ended. Younas *et al*. [165] highlighted the substantial influence of reservoir compaction on surface/subsurface subsidence, underscoring the need to answer critical questions related to this influence.

Several specific studies have been conducted of the subsidence associated with technologies related to the green energy transition. Rutqvist [104] showed that, assuming a Poisson ratio of 0.2, a Young’s modulus of 6 GPa, a reservoir thickness of 20 m, a reservoir depth of 2 km, and a pressure drawdown of 10 MPa, the approximately 3 cm of subsidence that had been measured above injection wells at the In Salah carbon sequestration site in Algeria was entirely consistent with the predictions of the classical analytical subsidence model developed by Geertsma [166]. Teatini *et al*. [160] used a poroelastic finite-element simulator to model the surface displacement due to cyclic injection/withdrawal of gas in the Lombardia field in northern Italy and were able to reproduce the vertical displacements, of the order of 8−10 mm, that were measured between 2003 and 2007. Jeanne *et al*. [161] reported an extensive modelling and monitoring programme of subsidence above the EGS at The Geysers geothermal field in northern California. Surface displacements were measured over an area of 132 km^2^, using synthetic aperture radar. They found that it was crucial, and challenging, to mathematically disentangle the surface displacement due to fluid injection/withdrawal, from the displacements due to rainfall and natural tectonic activity.

### 4.4. Induced seismicity

**Figure 6.**
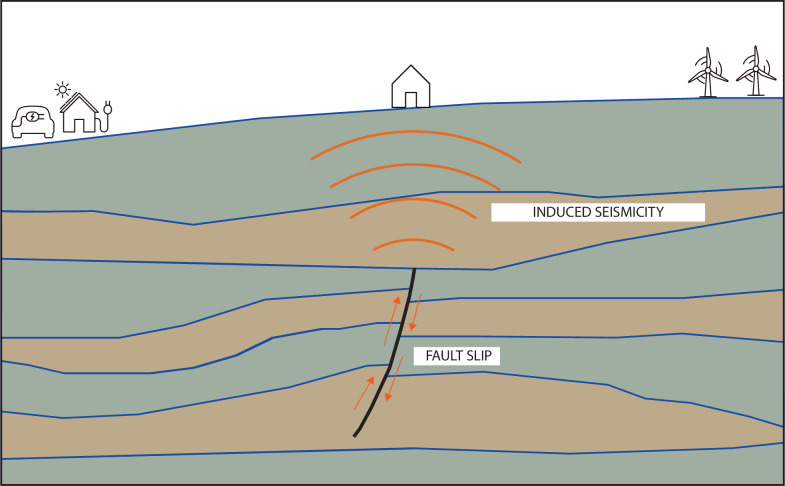
Subsurface stress changes due to fluid injection, extraction or migration can cause pre-existing faults to slip, thereby generating induced seismic events. These events may be felt at the surface and need to be prevented through operational constraints, subsurface rock and fluid characterization and proper site selection.

Injection of fluids into the subsurface causes an increase in pore fluid pressure, which in turn changes the effective stress that acts on existing faults and fractures. In general, an increase in pore pressure brings a fault closer to the state of stress at which slip will occur, potentially causing an abrupt release of energy known as a seismic event (figure 6). This type of seismicity is known as *induced seismicity*, or *fluid-induced seismicity* [108,167], in contrast to the natural seismicity that occurs along some faults without any influence of man-made fluid injection.

Schultz *et al*. [168] reviewed the state of knowledge of hydraulic fracturing-induced seismicity, highlighting the importance of addressing the unanswered questions in this area to mitigate potential risks. Verdon *et al*. [169] stressed the importance of differentiating between seismicity induced by industrial activities and that which arises through natural geological process, highlighting the need to address historic cases of induced seismicity and identify the unanswered questions in this area.

Major unanswered questions about subsurface geomechanics related to geothermal energy utilization revolve around understanding the uncertainties and risks associated with deep sedimentary and crystalline geologic systems [170]. These uncertainties translate into challenges for underground thermal energy storage, CO_2_ sequestration [171] and seasonal hydrogen storage [128]. One of the challenges in subsurface characterization is identifying highly heterogeneous permeability fields such as occur in geothermal reservoirs containing fracture networks [172]. The impact of geothermal energy extraction on groundwater temperature and quality, as well as the operational and geological controls of coupled poroelastic stresses and pore-pressure accumulation along faults, are also areas of concern with regard to induced seismicity [173].

Furthermore, subsurface engineering during geothermal operations tends to generate unintended fluid-flow paths, mechanical discontinuities, micro-seismic events and tremors [117]. The lack of borehole cores for DGE source characterization poses another significant challenge [174]. Understanding the stratification characteristics of subsurface rock structures and the impact of shallow geothermal energy use on groundwater temperatures are also relevant for geothermal energy development [175]. Moreover, understanding the changes in fracture properties, such as aperture and roughness, as a result of contact with acidic fluids, is essential for various subsurface activities, including geothermal energy extraction [176].

### 4.5. Caprock integrity, fluid leakage and migration

Geological storage of fluids requires effective sealing mechanisms, typically comprising one or more impermeable layers of rock that serve as a barrier against fluid migration. The specific configuration of the seal varies, depending on the nature of the stored fluid and the associated storage context. In fluid storage applications such as hydrogen storage or carbon sequestration, the seal typically resides above the formation, acting as a barrier to prevent the escape of fluids such as hydrogen or CO_2_ [177]. Differentiating between seals and caprocks is important, as caprocks serve as primary barriers against fluid migration in subsurface storage applications. The term ‘caprocks’ specifically refer to the impermeable layers directly above a reservoir, whereas the term ‘seals’ encompass any barriers that restrict fluid movement within or around the storage formation.

**Figure 7.**
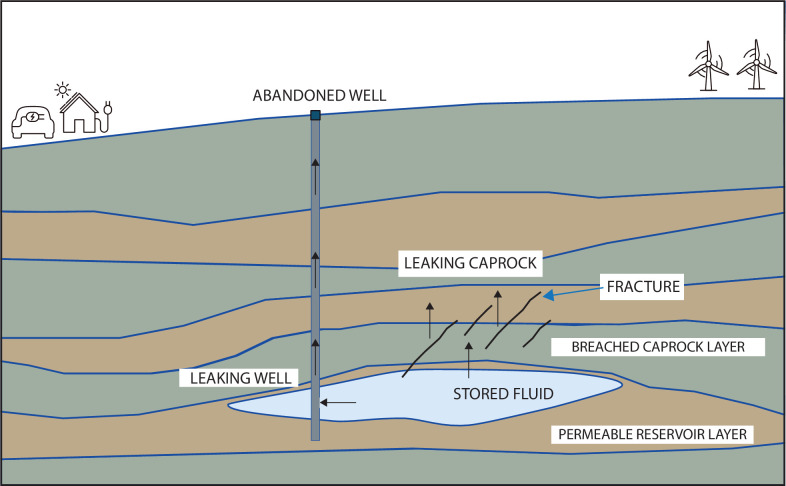
Stored fluids may leak back to the surface through abandoned and improperly sealed wells or through breached caprocks. Fluid migration can potentially be contained by hierarchical seals and caprocks, but may migrate into compromised abandoned wells or permeable fractures and faults located far from the injection site. Leakage can be managed through the sealing of wells, along with proper operational constraints and site selection.

For hydrogen storage, the seal may also comprise the laterally surrounding rocks, whereas in geological waste disposal scenarios, the seal encompasses the host rock itself, serving as the natural barrier that surrounds the stored waste [178]. The integrity of caprock, a specific type of sealing layer, assumes particular significance in the context of CO_2_ sequestration, due to its role in preventing CO_2_ or brine migration into shallow groundwater formations, thereby averting potential environmental contamination [179]. Understanding the nuanced interplay between fluids and sealing lithologies is essential for designing effective containment strategies and ensuring the long-term safety and security of subsurface storage operations (figure 7).

The long-term integrity of subsurface seals is of paramount importance for containing fluid migration and preventing environmental contamination. In the context of fluid storage, the objective is to impede the escape of stored fluids from the reservoir, whereas in the context of nuclear waste disposal, the goal is to prevent radioactive-laden fluids from migrating beyond the confines of the geological disposal facility. The interaction between fluids and sealing lithologies is complex, with certain rock layers exhibiting greater hindrance to fluid migration than others. Fractures and faults further complicate the sealing dynamics, as they may serve either as barriers or as migration pathways, contingent upon their stress state and their infilling characteristics.

To predict and prevent caprock integrity challenges in geomechanics, particularly for shales, sandstones and granites, it is essential to consider the geomechanical response of caprocks to CO_2_ intrusion, mineral alteration and injection-induced deformation. The potential for fault reactivation and seismic events should also be evaluated [180,181]. The influence of stress changes on caprock porosity, permeability and ground displacement can be studied using coupled hydro-geomechanical models [182,183]. Furthermore, the long-term variations of mineralogical and hydraulic properties of the caprock must be understood, in order to assess caprock integrity [184]. Carbon dioxide has an impact on the surface chemistry and chemical structure of shale caprocks, with important implication for sealing integrity [185]. Moreover, the influence of CO_2_ on seismic and ultrasonic properties of caprocks must be investigated, in order to understand its effect on caprock integrity [186].

The use of coupled multi-phase fluid flow and geomechanical modelling can provide insights into geomechanical changes, such as injection-induced fracture reactivation, which could lead to enhanced permeability and CO_2_ migration across caprock formations [13]. Furthermore, the impact of *in situ* stress and fault reactivation on seal integrity should be assessed to understand the lithology’s role in affecting seal integrity [180]. It is also important to consider the geomechanical characterization of caprock-reservoir systems, to estimate the spatial variation of *in situ* stress magnitude and orientation, which is necessary for evaluating practical storage capacity for geologic CO_2_ storage [187]. Long-term integrity of CO_2_ reservoir caprocks can be monitored by conducting time-lapse seismic imaging to map CO_2_ movement in the subsurface, as CO_2_ migration into the caprock might change its properties and impact its integrity [186,188].

Rock–fluid interactions, such as CO_2_-acidified brine flow, can modify the properties of the caprock and weaken the seal layer [176,189]. Hassanpouryouzband *et al*. [190] emphasized the potential of hydrogen to alter reservoir and caprock porosity and permeability, posing a threat to storage integrity. In the context of geological hydrogen storage, the reactivity of hydrogen with sandstone reservoirs has been a subject of investigation, with findings indicating potential alterations to reservoir and caprock properties [191,192]. Similarly, Szott and Miłek [193] discussed the risks associated with distinct leakage pathways, including migration to the caprock via activated fractures, in the context of CO_2_ sequestration.

## 5. Environmental impacts and social perception

Public concerns in the context of subsurface activities within the green energy transition encompass several aspects, particularly focusing on environmental and social implications. One major concern is induced seismicity, where there is significant anxiety about the possibility of subsurface activities, such as shale gas extraction and geothermal energy production, inducing seismic events, with induced earthquakes perceived more negatively than natural ones [194]. Environmental impacts, including water contamination from chemicals used in hydraulic fracturing and disruption to local ecosystems, are also critical issues. Health and safety concerns revolve around potential risks to nearby communities from air and water pollution, and the safety of emerging technologies such as hydrogen storage in geological formations [152,195]. Trust in regulatory oversight and the effectiveness of stringent regulations is crucial, as seen in the case of shale gas extraction in the UK, where even stringent regulations were met with public scepticism [196]. Effective risk communication and public engagement are essential, with studies indicating a preference for detailed, quantitative risk communication—although highlighting uncertainty can have the effect of increasing anxiety [197,198]. Economic and social impacts, such as concerns about ‘who benefits’ from subsurface activities and ‘who bears the risks’, also play a significant role, with perceptions of private companies profiting at the expense of public safety leading to opposition [194]. Social implications, including potential displacement, noise and disruption to local communities, underscore the importance of ensuring that local communities have a voice in the decision-making processes [199,200].

The societal perception of the role of subsurface geomechanics in the green energy transition is influenced by a range of values, beliefs and social contexts, as well as the inherent characteristics of the technologies themselves [201]. Effective communication and engagement strategies are essential for gaining societal support, particularly for emerging technologies such as geological hydrogen storage, which presents challenges in perceived safety and risk management [195]. Public acceptance of renewable energy technologies involves multiple dimensions such as socio-political, community and market acceptance. Studies have underscored the need to address location-related factors and community acceptance to promote cleaner energy production [202]. Understanding public responses and their drivers is crucial, since public support significantly affects the adoption and deployment of new technologies [203].

Induced seismicity is a major public concern associated with subsurface geomechanics. McComas *et al*. [194] found that the public perceives induced earthquakes more negatively than natural ones, and attributing benefits such as the provision of renewable energy does not enhance their acceptability. Effective risk communication formats that combine qualitative and quantitative information with risk comparisons are more effective, but including uncertainty and expert confidence statements can increase public concerns [197,198].

Studies on shale gas and geothermal energy provide valuable insights into public perception. Evensen *et al*. [196] studied how the public perceives induced seismicity and its influence on regulatory changes. Their study, which involved a longitudinal experimental survey in the UK, aimed to determine whether framing and information provision affect public support for changing regulations on shale gas extraction. The surveys explored various framing techniques, including quantitative versus qualitative framings, information about regulatory limits in other countries and the comparison of seismicity from different industries. Despite these efforts, the study found that public support for altering existing policies remained low, and the framing of information had minimal impact on these perceptions. The primary factor influencing negative public reactions was the type of activity causing the seismic events, with shale gas extraction eliciting the most adverse responses. These findings highlight the challenges in gaining public acceptance and the crucial role of transparent and effective communication strategies in the context of energy policy and induced seismicity [196].

Comparatively, risk communication about DGE is perceived as more trustworthy and less concerning, indicating that the framing of technology may significantly influence public attitudes [197]. Trutnevyte and Ejderyan [198] tested three risk communication formats with 590 participants and found that formats including quantitative data and risk comparisons were preferred for their clarity. However, adding uncertainty and expert confidence reduced clarity and increased concern. Similarly, Knoblauch *et al*. [197] also found that quantitative and comparative risk communication formats were better received, but noted a trade-off between transparency and increased public anxiety. Both studies concluded that although detailed risk information is valued, it can also raise concerns, particularly with shale gas as compared with DGE.

Trust in actors involved in industrial processes is crucial for public acceptance. For example, the Groningen gas field in The Netherlands experienced significant public concern due to increased earthquake activity from gas extraction. Effective risk management and communication strategies are necessary to restore public confidence and reduce anxiety [200]. Drawing lessons from CO_2_ and natural gas storage can inform strategies for geological hydrogen storage, underscoring the need for site-specific communication to address societal concerns effectively [195]. Scientific knowledge and perceived knowledge insufficiency are critical factors in shaping risk perception. Kahlor *et al*. [204] found that risk information avoidance intentions are influenced by subjective norms, attitudes and perceived knowledge insufficiency. Additionally, Pidgeon et al. [205] demonstrated that public perception of geoengineering technologies is influenced by baseline awareness, concern about climate change and perceived effectiveness, cost and risks of the technologies.

Despite the advancements in understanding public perception and risk communication, gaps remain. For instance, there is a need for test facilities to aid the design of low-cost, high-resolution, unobtrusive seismic monitoring in seismically noisy urban environments and dedicated through-fault-zone test sites to understand fault transmissivity and reactivation [201]. Additionally, more empirical research is required to explore how communication and information provision on induced seismicity influence perceptions, beliefs, and acceptance of associated technologies [198].

## 6. Summary and conclusions

This article has presented a review of key challenges and advances in the field of subsurface geomechanics in the context of the green energy transition, a topic both technically challenging and socially complex, yet crucial for the sustainable deployment of renewable energy technologies at a suitably large scale.

Subsurface geomechanics involves the understanding of rock deformation, fault mechanics and coupled fluid–rock interactions, particularly under the high-pressure, high-temperature conditions that are typical of subsurface environments. Numerical, experimental and mathematical advancements are pivotal for predicting and mitigating risks such as induced seismicity, wellbore instability and fluid leakage, which are critical for ensuring the safety and efficacy of subsurface energy technologies. The integration of state-of-the-art field monitoring techniques, such as seismic monitoring, with numerical techniques for geomechanical modelling, and laboratory experiments, has enhanced our ability to simulate and understand the complex processes occurring in the subsurface.

There is an ongoing cross-disciplinary effort to develop the required expertise across all applications, with key central dependencies on the understanding of the fundamental behaviour of fluid and rocks under coupled thermo-poro-mechanical conditions. These advances are important in the context of developing next-generation predictive capabilities, while also better informing regulatory practices that will environmentally shield, while at the same time support, the development of a thriving energy industry, contributing more effectively to the management of subsurface resources over generations to come.

This article has also highlighted some additional significant challenges in the context of public perception and social acceptance of subsurface activities. Induced seismicity emerges, in particular, as a primary public concern, in the general context of fluid extraction and geothermal energy, as induced earthquakes tend to be perceived more negatively than natural ones. This underscores the importance of effective risk communication, public trust in regulatory oversight and the need for transparent, quantitative communication strategies that address public concerns while avoiding unnecessary alarm.

The case of shale gas in the UK illustrates that even with stringent regulations, public scepticism can persist, particularly when there is a perception that the risks are not equitably distributed, or that private companies are profiting at the expense of public safety. The review also emphasized the socio-economic and environmental implications of subsurface activities, including issues such as potential water contamination, disruption to local ecosystems and the broader impact on community well-being, all of which are central to public concerns.

Addressing these issues requires not only technological solutions but also inclusive decision-making processes that engage local communities and consider their values and concerns. Studies have shown that public acceptance of renewable energy technologies is multi-faceted, involving socio-political, community and market acceptance. Effective public engagement strategies are essential for fostering the necessary societal support for the deployment of subsurface technologies, particularly as they relate to the green energy transition.

As the world shifts towards more sustainable energy systems, the subsurface will play an increasingly important role in providing the necessary resources and storage capacities. The effective management of subsurface processes is essential for the success of technologies such as geothermal energy production, carbon sequestration, nuclear waste disposal or hydrogen storage and subsequent utilization for electricity production. However, the technical challenges must be matched by efforts to address social concerns and build public trust. The integration of advanced geomechanical research with proactive, transparent and inclusive public engagement strategies will be key to overcoming these challenges and realizing the full potential of subsurface technologies in the green energy transition. This dual focus on technical excellence and social responsibility will ensure that subsurface geomechanics not only advances scientific understanding but also contributes meaningfully to a sustainable and socially equitable energy future.

## Data Availability

This article has no additional data.
